# Establishment of a TaqMan Quantitative Real-Time PCR for Detecting *Lawsonia intracellularis*

**DOI:** 10.3390/vetsci12050450

**Published:** 2025-05-08

**Authors:** Zhiqiang Hu, Ranran Lai, Wei Xu, Ran Guan, Zhimin Zhang, Guangwen Yan, Guiying Hao

**Affiliations:** 1 Key Laboratory of Animal Epidemic Disease Detection and Prevention in Panxi District, College of Animal Science, Xichang University, Xichang 615013, China; zhiqianghu0624@xcc.edu.cn (Z.H.); guanran@xcc.edu.cn (R.G.); 18215755808@163.com (Z.Z.); ygwdky@126.com (G.Y.); 2 Shandong New Hope Liuhe Agriculture and Animal Husbandry Technology Co., Ltd., Dezhou 253034, China; lairanran18@163.com; 3 MOA Key Laboratory of Animal Virology, Center for Veterinary Sciences, Department of Veterinary Medicine, College of Animal Sciences, Zhejiang University, Hangzhou 310058, China; xuwei88@zju.edu.cn

**Keywords:** porcine proliferative enteropathy, *Lawsonia intracellularis*, TaqMan-qPCR, clinical detection

## Abstract

Porcine proliferative enteropathy (PPE) is an infectious disease in pigs, caused by *Lawsonia intracellularis* (LI), affecting their intestines during growth and finishing stages, leading to higher production costs. Current detection methods for LI face two main challenges, delayed results and high costs, making them impractical for large-scale pig farming epidemiological surveys. This study developed a TaqMan-qPCR method using specific probes and primers based on the LI aspartate ammonia lyase genes from GenBank, completing detection in just 45 min. After optimizing reaction conditions, sensitivity analysis revealed that the detection limit of this method was 4.6 copies/μL targeting standard plasmids. The results of the specificity analysis showed no cross-reactivity with other common porcine pathogens, highlighting its specificity. The inter- and intra-group coefficients of variation were both <1%, indicating high reproducibility. Furthermore, the TaqMan-qPCR demonstrated 100% relative sensitivity, and a 92.50% compliance rate compared to conventional PCR, suggesting it could be a complement to the conventional PCR method. In summary, the TaqMan-qPCR method established in this study is not only suitable for epidemiological investigations and early qualitative and quantitative diagnosis of proliferative enteropathy in pigs, but it is also valuable for studying the biological characteristics of LI.

## Simple Summary

Porcine proliferative enteropathy (PPE) is a costly intestinal disease in pigs caused by *Lawsonia intracellularis* (LI) during their growth stages. Existing LI detection methods are slow and expensive, limiting their use in large-scale pig farming. This study develops a TaqMan-based quantitative real-time PCR (TaqMan-qPCR) method for LI detection, validated for its high sensitivity, specificity, and reliability. It is ideal for epidemiological studies, early diagnosis, and understanding LI’s biological traits.

## 1. Introduction

*Lawsonia intracellularis* (LI) is a strictly intracellular parasitic Gram-negative bacterium belonging to the *Enterobacteriaceae* family [1,2]. It is the pathogen of proliferative enteropathy (PPE), primarily parasitizing the intestines of pigs, causing neoplastic-like proliferation of immature intestinal epithelial cells in the ileum, cecum, and colon, which leads to thickening of the intestinal mucosa and functional disorders [3,4,5]. LI primarily infects pigs during the growing and finishing stages, presenting in acute, chronic, or subclinical forms, with the chronic form being the most prevalent. Clinical manifestations include intermittent diarrhea, reduced appetite, impaired growth, and diminished feed efficiency, all of which contribute to elevated production costs [6]. Consequently, LI is recognized as an economically significant disease and a major factor impeding weight gain in finishing pigs [7,8,9]. Initially identified in 1931, LI has since become endemic on a global scale [10,11,12]. Although data on the prevalence of LI in China are limited, recent years have witnessed a detection rate as high as 93.6% in some large-scale pig farms, coinciding with the intensification level of swine production [13]. Thus, improving the rapid detection of LI is essential for the effective prevention and management of PPE.

At present, laboratory diagnosis of LI can be achieved through pathological histology or specific immunofluorescence staining techniques, such as Warthin–Starry silver staining, Ziehl–Neelsen staining, indirect immunofluorescence assay (IFA), and immunohistochemistry (IHC) [14]. However, these methodologies are limited to postmortem detection and are not applicable for live animal testing, thereby constraining the evaluation of infection status and severity within pig populations. Furthermore, while enzyme-linked immunosorbent assay (ELISA) and immunoperoxidase monolayer assay (IPMA) are available for monitoring live infections of LI, their high costs render them impractical for large-scale routine surveillance [15,16].

In recent years, the intensification of swine production has led to the widespread adoption of qPCR as the predominant method for pathogen detection in large-scale pig farms. Consequently, this study involved the design of specific primers and TaqMan probes targeting the conserved region of the aspartate ammonia lyase (aspA) gene of LI, which encodes aspA enzyme. The aspA enzyme catalyzes the reversible conversion of L-aspartate to fumarate, releasing ammonia [17], which plays an important role in bacterial growth and pathogenesis [18,19]. Then, we developed an effective detection method for LI utilizing real-time fluorescence quantitative PCR technology, which was subsequently applied in clinical settings. The establishment of this method offers a scientific foundation for the rapid diagnosis and timely prevention and control of LI in clinical contexts.

## 2. Materials and Methods

### 2.1. Primers and Probes

The aspA gene sequence of LI (Gene ID CP004029.1, AM180252.1, and CP107054.1) from GenBank was analyzed using DNAStar software (Version 7.0) to design specific primers and a TaqMan probe with Primer Express 3.0, focusing on a conserved sequence region (Table 1). The resulting amplified gene fragment measures 158 base pairs in length. As outlined in Table 1, the probe is tagged with a FAM fluorescent reporter at the 5′ end and a BHQ1 quencher at the 3′ end. Both the primers and the probe were synthesized by Sangon Biotech (Shanghai) Co., Ltd. (Shanghai, China).

### 2.2. Standard Plasmids

The pUC57-LI plasmid was constructed by synthesizing and cloning amplified sequences of the aspA gene (Gene ID: CP004029.1, AM180252.1, and CP107054.1) in GenBank into the pUC57 vector. Quantification of the standard plasmid was performed using a UV–visible spectrophotometer, and copy numbers were calculated using a designated formula [20]. The plasmids were then subjected to serial dilutions at a 10-fold scale (concentrations ranging from 4.6 × 10^0^ to 4.6 × 10^9^ copies/μL) and stored at −20 °C for future use.

$$Plasmid\;copies/\mu L=\frac{\left(6.02\times 10^{23}\right)\times \left(X\;\mathrm{ng}/\mu L\times 10^{-9}\right)}{plasmid\;length\left(\mathrm{bp}\right)\times 660}$$

### 2.3. Optimization of Reaction Conditions

Primer and probe concentrations were systematically optimized using a matrix approach in a 20 μL reaction volume with AceQ Universal U + Probe Master Mix V2 (Vazyme Q513, Vazyme, Nanjing, China). Primer concentrations of 10 μM (0.2 to 0.8 μL) and probe concentrations of 10 μM (0.1 to 0.4 μL) were tested with annealing temperatures from 55 °C to 61 °C. The primary aim was to minimize the quantification cycle (Cq) value while maximizing the increase in fluorescence intensity (ΔRn), thereby enhancing the amplification efficiency and sensitivity of the reaction.

### 2.4. Evaluation of Sensitivity and Construction of Standard Curves

Amplification was performed using a standard plasmid template subjected to 10-fold serial dilutions, with concentrations ranging from 4.6 × 10^9^ to 4.6 × 10^0^ copies/μL under optimized conditions. The resulting amplification kinetic curves were analyzed, and a standard curve for the established TaqMan-qPCR method was constructed by plotting the logarithm of the copy number of the positive standard plasmid on the x-axis against the Cq values on the y-axis. This analysis yielded the standard linear regression equation for the method.

### 2.5. Evaluation of Specificity

To assess the specificity of the TaqMan-qPCR method, it was employed to detect the cDNA of eight prevalent porcine viruses, namely Classical Swine Fever Virus (CSFV), Porcine Epidemic Diarrhea Virus (PEDV), Transmissible Gastroenteritis Virus (TGEV), Rotavirus (RV), Porcine Reproductive and Respiratory Syndrome Virus (PRRSV), Pseudorabies Virus (PRV), Porcine Parvovirus (PPV), and Porcine Circovirus Type 2 (PCV2), as well as three common porcine intestinal bacteria: *Escherichia coli* (*E. coli*), *Salmonella enteritidis* (*SE*), and *Brachyspira hyodysenteriae* (*B. h*). The pUC57-LI standard plasmid was utilized as the positive control, while double-distilled water (ddH_2_O) served as the negative control. All cDNA templates tested positive before the experiment, and the corresponding Cq values for each pathogen were between 20 and 30. The concentration of the pUC57-LI standard plasmid was 4.6 × 10^5^ copies/μL.

### 2.6. Evaluation of Reproducibility

The developed TaqMan-qPCR method was executed using pUC57-LI standard plasmids at concentrations of 4.6 × 10^5^, 4.6 × 10^3^, and 4.6 × 10^1^ copies/μL as templates, with each concentration tested in triplicate under optimized reaction conditions. Each experimental batch comprised three replicates for each dilution level, and the resulting Cq values were subjected to statistical analysis to determine intra- and inter-group coefficients of variation (CV). This analysis facilitated the assessment of the method’s reproducibility and stability.

### 2.7. Comparison Between TapMan-qPCR and Conventional PCR with Clinical Samples

Clinical samples, including bloody stool (N = 20), anal swabs (N = 16), and environmental samples (N = 4) from LI-positive farms, were collected and analyzed using both the established TaqMan-qPCR method and the conventional PCR technique. The performance of the established TaqMan-qPCR method was assessed against the conventional PCR method by calculating relative sensitivity as [(true positive/(true positive + false negative)] × 100% and relative specificity [(true negative/(true negative + false positive)] × 100% [21].

## 3. Results

### 3.1. Optimization of Reaction Conditions

Optimized via the matrix method, the reaction conditions included an initial 2 min incubation at 37 °C and a 5 min denaturation at 95 °C, followed by 40 cycles of 95 °C for 10 s and 60 °C for 30 s. The optimal 20 μL reaction volume included 10 μL of 2 × AceQ Universal U + Probe Master Mix V2, 0.4 μL each of 10 μmol/L forward and reverse primers, 0.2 μL of 10 μmol/L probe, 1 μL of template, and deionized water to complete the volume.

### 3.2. Establishment of the Standard Curve

**Figure 1:**
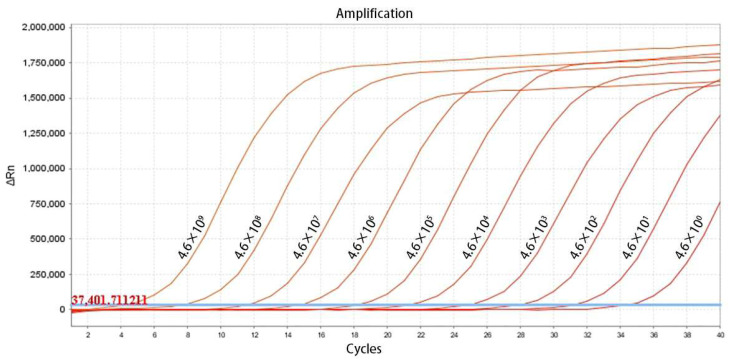
Amplification curve of the TaqMan-qPCR method for detection of LI.

**Figure 2:**
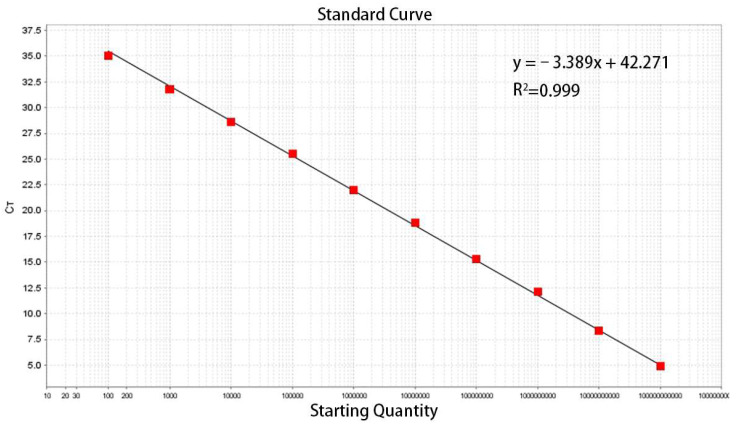
Standard curve of the TaqMan-RT-PCR method for detection of LI.

The TaqMan-qPCR assay effectively detected standard plasmids across concentrations from 4.6 × 10^9^ to 4.6 × 10^0^ copies/μL, showing a strong linear correlation. The minimum detectable concentration was 4.6 × 10^0^ copies/μL (Figure 1). As illustrated in Figure 2, the derived standard curve equation was y = −3.389x + 42.271, with a coefficient of determination (R^2^) of 0.999 and an efficiency (Eff%) of 96.0%.

### 3.3. Specificity Testing

**Figure 3:**
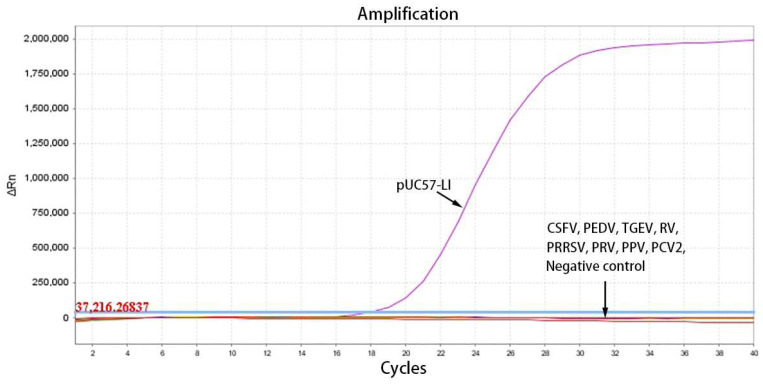
The amplification curves of specificity testing within eight porcine viruses.

**Figure 4:**
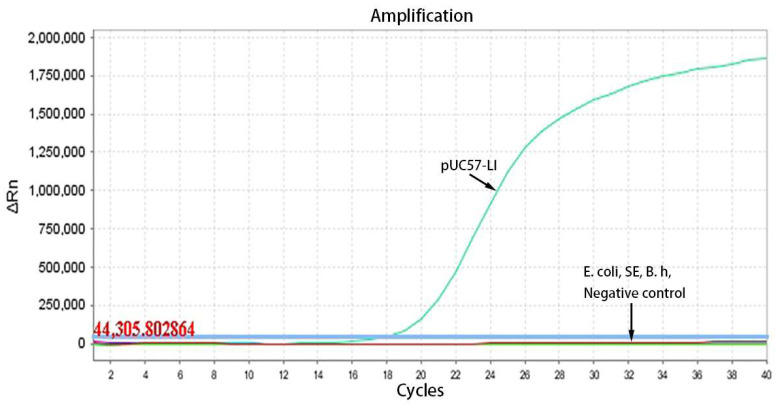
The amplification curves of specificity testing within three porcine intestinal bacteria.

The optimized reaction protocol was utilized to detect cDNA from a range of porcine pathogens, encompassing both viruses and bacteria. As illustrated in Figure 3, the specific amplification curve was observed exclusively for the pUC57-LI standard plasmids, with no amplification curves detected for the other common viruses (CSFV, PEDV, TGEV, RV, PRRSV, PRV, PPV, PCV2). Similarly, as depicted in Figure 4, the specific amplification curve was present only for the UC57-LI standard plasmids, while no amplification curves were observed for the bacterial samples (*E. coli*, *SE*, and *B. h*). These findings indicate that the TaqMan-qPCR assay demonstrates high specificity, with no cross-reactivity observed with common porcine pathogens.

### 3.4. Repeatability Testing

As demonstrated in Table 2, the intra-group CV ranged from 0.24% to 0.74%, while the inter-group CV ranged from 0.37% to 0.69%, indicating the method’s excellent reproducibility.

### 3.5. Comparison Between the TaqMan-RT-PCR Method and the Conventional PCR Method with Clinical Samples

As demonstrated in Table 3, when samples were analyzed using both the TaqMan-qPCR and the conventional PCR method, the TaqMan-qPCR exhibited a relative sensitivity of 100%, signifying the same sensitivity in comparison to the conventional PCR method. The relative specificity of the TaqMan-RT-PCR was 50.00%, indicating enhanced specificity over the conventional PCR method. Additionally, the compliance rate for the TaqMan-RT-PCR method was 92.50%, suggesting that the TaqMan-RT-PCR method could potentially serve as a substitute for the conventional PCR method.

## 4. Discussion

At present, qPCR technology is extensively utilized in disease diagnostics within intensive pig farming due to its wider applicability, rapid processing, and high sensitivity. Nevertheless, the application of qPCR technology for the clinical detection of LI remains underexplored [3,4,22,23]. A particular study assessed the efficacy of the IHC method for detecting LI in 165 intestinal sections, confirming positive results in 33 samples using both PCR and IHC methodologies, highlighting the potential of PCR in diagnosing LI compared to traditional detection methods [24]. Additionally, research conducted by Mirjam Arnold et al. employed qPCR technology to detect LI in European pig farms, providing not only qualitative diagnostic results but also precise bacterial load measurements. This facilitated the evaluation of the relationship between bacterial loads and LI transmission characteristics in pig farms, further emphasizing the significant role of qPCR in the clinical diagnosis of LI [25]. Moreover, studies have demonstrated the potential of qPCR in detecting LI infections in wildlife and stray cats, thereby establishing the application value of qPCR for LI detection in epidemiological investigations [26]. Collectively, these studies underscore the considerable utility of qPCR in the surveillance and management of LI infections in pig herds.

In this study, primers and TaqMan probes were meticulously designed using the conserved regions of the aspA gene from LI sequences archived in GenBank. The detection process was completed in a mere 45 min, offering a more convenient and time-efficient alternative to traditional PCR methodologies, while also exhibiting enhanced sensitivity. H. Nathues and K. Holthaus developed an RT-PCR technique with a detection sensitivity of a single copy per reaction; however, reliable quantification was achieved within the range of 10^1^ to 10^7^ copies per μL of reaction volume [23]. Recently, Ren et al. developed a multiplex pathogen diagnostic technique that includes the detection of LI, achieving a detection limit of 10 copies/μL [27]. The TaqMan-qPCR method developed in the present study demonstrated a detection limit of 4.6 copies/μL, indicating enhanced sensitivity compared to previously reported methodologies. Furthermore, Ken Steen Pedersen et al. devised a qPCR method for the detection of LI in fecal samples, with a detection limit of 4.8 log10 LI bacteria per gram of feces [28]. Additionally, the TaqMan-qPCR method developed in this study demonstrated suitability for a variety of sample types, including bloody stool, anal swabs, and environmental samples, thereby offering a broader range of application scenarios. Comparative testing results further indicated that the developed TaqMan-qPCR method serves as a complement to the conventional PCR method.

## 5. Conclusions

In conclusion, the TaqMan-qPCR assay developed in this study for the detection of LI represents a significant advancement in the diagnosis of PPE. This method demonstrates superior analytical sensitivity, with the detection limit of 4.6 copies/μL, while maintaining 100% relative sensitivity and achieving a 92.50% compliance rate compared to conventional PCR techniques, thereby affirming its reliability in veterinary diagnostic contexts. The assay’s operational reliability is further substantiated by its stringent specificity profile, which shows no cross-reactivity with either common porcine viruses or bacteria, alongside its high reproducibility, as indicated by inter- and intra-group CVs below 1%. These combined characteristics underscore the technical superiority of this approach for field applications, providing a theoretical foundation for the development of targeted prevention and control strategies against LI infection.

## Figures and Tables

**Table 1:** Primers and probes.

Primer or Probe	Sequence (5′ to 3′)
F	GGTATTGGTATTCTCCTTTCTCA
R	CTGCATGTAATGAAATCATAAATG
Probe	FAM-TGTTGTGGATTGTATTCAAGGAGGTG-BHQ1

**Table 2:** Intra-assay and inter-assay reproducibility test of the TaqMan PCR.

Concentration of Standard Plasmids (Copies/μL)	Intra-Coefficient of Variation			Inter-Coefficient of Variation		
	n	$\bar{X}$ ± SD	CV%	n	$\bar{X}$ ± SD	CV%
4.6 × 10^5^	3	18.34 ± 0.04	0.48%	3	18.24 ± 0.09	0.52%
4.6 × 10^3^	3	24.75 ± 0.06	0.24%	3	24.84 ± 0.09	0.37%
4.6 × 10^1^	3	31.49 ± 0.20	0.74%	3	31.63 ± 0.22	0.69%

**Table 3:** Comparison between the TaqMan-RT-PCR method and the conventional PCR method with clinical samples.

		Conventional PCR		Total
		+	−	
TaqMan-RT-PCR	+	34	3	37
	−	0	3	3
Total		34	6	40
*Relative sensitivity = 34/34 = 100%*				
*Relative specificity = 3/6 = 50.00%*				
*Compliance rate = 37/40 = 92.50%*				

## Data Availability

No datasets were generated or analyzed during the current study.

## Abbreviations

The following abbreviations are used in this manuscript:

PPE	porcine proliferative enteropathy
LI	Lawsonia intracellularis
IFA	indirect immunofluorescence assay
IHC	immunohistochemistry
ELISA	enzyme-linked immunosorbent assay
IPMA	immunoperoxidase monolayer assay
Cq	quantification cycle
CV	coefficients of variation
